# Reinfection of COVID-19 in Pakistan: A First Case Report

**DOI:** 10.7759/cureus.11176

**Published:** 2020-10-26

**Authors:** Muhammad Hanif, Muhammad Adnan Haider, Mukarram Jamat Ali, Sidra Naz, FNU Sundas

**Affiliations:** 1 Internal Medicine, Khyber Medical College, Peshawar, PAK; 2 Internal Medicine, Allama Iqbal Medical College/Jinnah Hospital, Lahore, PAK; 3 Internal Medicine, King Edward Medical University/Mayo Hospital, Lahore, PAK; 4 Internal Medicine, University of Health Sciences, Lahore, PAK

**Keywords:** covid-19, sars-cov-2, convalescent plasma, rt-pcr

## Abstract

Since its spread across the world, coronavirus disease 2019 (COVID-19) has posed a severe public health threat, and many unanswered questions about COVID-19 remain. Antibodies have been detected a few days after the onset of infection, and in some patients, these antibodies wane quickly. To date, it is unknown whether all infected patients induce an adequate protective immune response or how long this effect remains. Here, a first case report of COVID-19 reinfection in Pakistan is reported within two months of complete recovery from the first severe acute respiratory syndrome-coronavirus-2 (SARS-CoV-2) infection - confirmed with two sequential negative nasopharyngeal swabs.

## Introduction

Coronavirus disease 2019 (COVID-19), caused by the severe acute respiratory syndrome-coronavirus-2 (SARS-CoV-2), initially began in Wuhan, China, and now has become a global pandemic [1]. Zhao et al. reported that the median time from COVID-19 symptom onset to antibody detection is 12 days for immunoglobulin (Ig) M antibodies and 14 days for IgG antibodies, and it is unclear whether all patients mount a protective response and how long any protective impact will last [2]. Recently, two elderly patients with COVID-19 pneumonia presented with new milder symptoms, and a repeated nasopharyngeal swab test was positive for COVID-19 a few days after they were discharged with complete clinical remission and two consecutive negative nasopharyngeal swabs [3]. COVID-19 reinfection is possible in recovered patients. We report a unique case of a patient from Pakistan (a country hugely affected by the pandemic) who recovered from COVID-19 (confirmed with two consecutive negative nasopharyngeal swabs) and was reinfected with SARS-CoV-2 within two months of initial recovery.

## Case presentation

**Figure 1:**
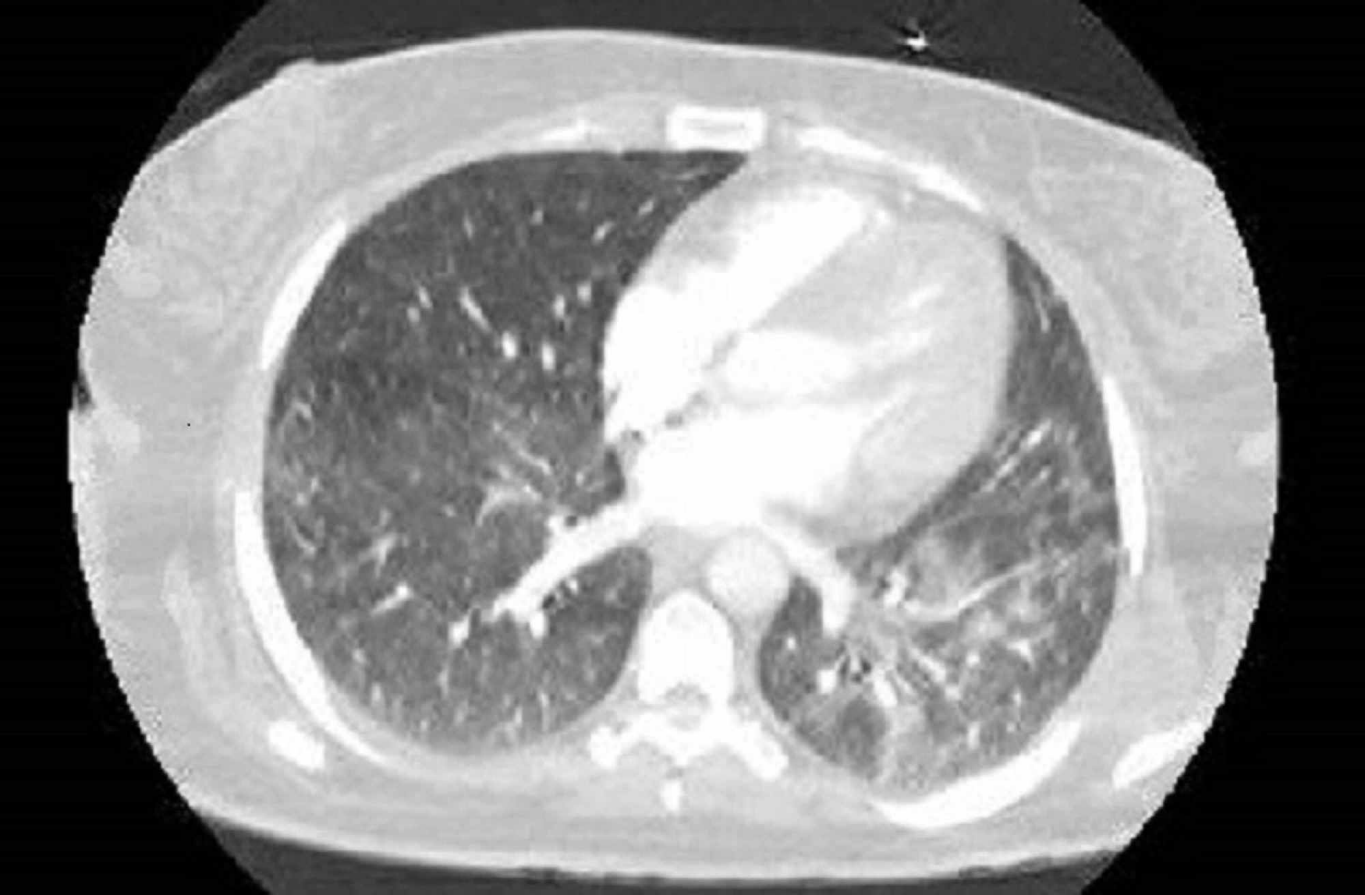
CT chest showing interstitial infiltrates

A 58-year-old cardiac surgeon with no significant past medical history was admitted to the hospital with concerns of fatigue, headache, and sore throat several days after performing coronary artery bypass grafting on two patients, who were later diagnosed as SARS-CoV-2 positive. On examination, his blood pressure was 125/85 mmHg, and his pulse was 76 beats/minute and regular. The rest of his examination findings were unremarkable. A nasopharyngeal swab test for COVID-19 was positive on quantitative reverse-transcriptase-polymerase-chain reaction (qRT-PCR) assay on April 25, 2020. A computed tomography scan of the chest revealed findings compatible with interstitial pneumonia (Figure 1). Treatment with oxygen supplementation and intravenous azithromycin was started. The patient was isolated during the entire hospitalization period, then discharged upon resolution of infection on two consecutive negative nasopharyngeal swabs after staying in the hospital for one week. Approximately two months later, on June 19, 2020, the patient developed a fever (>39°C), headache, and muscle aches after reexposure to patients with COVID-19 during cardiac surgery. A COVID-19 nasopharyngeal swab was again positive on polymerase-chain reaction (PCR), and blood tests were normal regarding both inflammation and respiratory parameters. He was vitally stable and quarantined for two weeks. Repeated PCR for SARS-CoV-2 was negative after two weeks.

## Discussion

Since the beginning of the pandemic, positive qRT-PCR tests for SARS-CoV-2 in patients with confirmed COVID-19 after negative results on two consecutive tests and improvement of their clinical symptoms have been published in the literature [4-7]. However, contrary to our case, these positive tests occurred shortly after the negative test results or patient discharge, and may not truly represent reinfection. In patients who tested positive on repeat ribonucleic acid (RNA) test after being cleared from isolation, coronavirus was not isolated in the cell culture of these patients [8]. Although the duration of viral RNA shedding is inconsistent, the longest median duration of viral RNA shedding as detected from nasopharyngeal specimens was 42 days [9].

On the other hand, there is a possibility that previous negative COVID-19 test results were false-negatives in those recovered patients due to limitations of test methods [10]. Fang et al. reported that 29% of COVID-19 patients who initially tested negative despite having typical COVID-19 symptoms eventually tested positive through serial testing [11].

Currently, it is unknown whether every infected patient mounts a protective immune response and how long any protective impact will last. Animal studies show that the immune response to infection may offer some protection against reinfection, at least temporarily. For example, in one animal study, all nine rhesus macaques, upon rechallenge with the same viral dose after 35 days, had an anamnestic response [12].

In our case, the patient who had recovered from COVID-19 two months previously later developed fever, headache, and muscle aches. Due to a high suspicion of COVID-19, his nasopharyngeal swab was sent for RT-PCR, which was positive. Unfortunately, there are some technical limitations of testing serum antibodies in Pakistan. Serologic screening will be an essential tool to help understand population immunity as the presence of antibodies is reflective of a protective immune response.

## Conclusions

It is critical to determine how long antibodies will last after SARS-CoV-2 infection. Our case may indicate a risk of reinfection in COVID-19 after full recovery. Antibodies may not last for a long period after infection. Thus, personal preventative and public health measures remain the primary preventative methods.
